# Scoping review of fidelity strategies used in behaviour change trials delivered in primary dental care settings

**DOI:** 10.1186/s13063-024-08659-9

**Published:** 2024-12-18

**Authors:** V. Lowers, R. Kirby, B. Young, R.V. Harris

**Affiliations:** Department of Public Health, Policy and Systems, Institute of Population Health, University of Liverpool, Whelan Building, Liverpool, L69 3GL UK

**Keywords:** Fidelity, Behaviour change, Primary dental care, Intervention implementation, Dental teams

## Abstract

**Background:**

Primary dental care settings are strategically important locations where randomised controlled trials (RCTs) of behaviour change interventions (BCIs) can be tested to tackle oral diseases. Findings have so far produced equivocal results. Improving treatment fidelity is posed as a mechanism to improve scientific rigour, consistency and implementation of BCIs. The National Institutes of Health Behaviour Change Consortium (NIH BCC) developed a tool to assess and evaluate treatment fidelity in health behaviour change interventions, which has yet to be applied to the primary dental care BCI literature.

**Method:**

We conducted a scoping review of RCTs delivered in primary dental care by dental team members (in real-world settings) between 1980 and 2023. Eligible studies were coded using the NIH BCC checklist to determine the presence of reported fidelity strategies across domains: design, training, delivery, receipt and enactment.

**Results:**

We included 34 eligible articles, reporting 21 RCTs. Fidelity reporting variations were found both between and within NIH BCC domains: strategy reporting ranged from 9.5 to 85.7% in design, 9.5 to 57.1% in training, 0 to 66.7% in delivery, 14.3 to 36.8% in receipt and 13.3 to 33.3% in enactment. The most reported domain was design (*M* = 0.45), and the least reported domain was delivery (*M* = 0.21). Only one study reported over 50% of the recommended strategies in every domain.

**Conclusions:**

This review revealed inconsistencies in fidelity reporting with no evidence that fidelity guidelines or frameworks were being used within primary dental care trials. This has highlighted issues with interpretability, reliability and reproducibility of research findings. Recommendations are proposed to assist primary dental care trialists with embedding fidelity strategies into future research.

**Supplementary Information:**

The online version contains supplementary material available at 10.1186/s13063-024-08659-9.

## Background

Oral diseases are a global health problem affecting as many as 3.5 million people worldwide [1]. This is despite them being largely preventable and often easily treatable if caught at an early stage [2]. Unfortunately, there are stark socioeconomic inequalities in the prevalence of oral diseases across the globe [3–5], so strategies to prevent and manage them are an important part of approaches for tackling health inequalities. Since oral health behaviours such as tooth brushing and sugary diets are key causative factors, behaviour change interventions have an important place within the armamentarium of disease prevention; primary care dentistry is an important setting where behaviour change intervention delivery has the potential to improve the oral health of individuals [6] and populations [7].

One intervention approach that has gained popularity is the use of behaviour change theory in the development of patient-level interventions [8]. Several studies have concluded that behaviour change techniques can be usefully applied to dental patient care [9–11]. However, randomised controlled trials (RCTs) of oral health targeted behaviour change interventions (BCIs) have produced equivocal results [12, 13]. Inconsistent reporting is often cited as a barrier to drawing meaningful conclusions from such studies; for example, both Adair and colleagues who studied BCIs in RCTs [14] and Werner and colleagues [15] found very small differences in effect size according to the oral health outcome measures used, and rated the certainty of evidence as low.

BCIs come in various guises, but they typically comprise multiple components and consist of multiple features [16]. Best practice in the development of BCIs involves the identification and implementation of theory-based ‘active ingredients’ that underpin the mechanisms that drive behaviour change [17]. To enable meaningful conclusions to be drawn from studies, it is important that BCIs are delivered as intended, with as much consistency as possible [18]. When considering the influence of variables such as the characteristics of those delivering interventions (i.e. personality, skill, warmth) or context dependent factors (especially important in multisite RCTs where there may be inconsistencies between sites), it is clear that achieving this consistency is a challenge within the BCI research paradigm.

To address these issues, increasing emphasis has been placed on treatment fidelity. The National Institutes of Health Behaviour Change Consortium (NIH BCC) defined treatment fidelity as ‘methodological strategies used to monitor and enhance the reliability and validity of behavioural interventions’ [19]. Fidelity strategies are concerned with monitoring, assessing and enhancing both adherence to intervention protocols as well as competency of intervention delivery [20], both of which are important aspects of providing increased confidence that effect sizes are due to the intervention alone [21, 22]. These strategies can enhance both internal validity and external validity [23], and where there is inadequate attention to fidelity, the interpretation of findings may be vulnerable to misappropriation and difficulties with replication important for real-world application [24].

The NIH BCC treatment fidelity framework [19] was developed to assist researchers in embedding fidelity strategies into studies, with a later version updated to include public health contexts [23]. The framework identifies five domains and associated strategies that should be considered:

**Table:** 

NIH BCC fidelity domain	Domain aims
1. Study design	Ensures study designs test their hypotheses in line with relevant underlying theoretical process
2. Interventionist training	Ensures standardised approaches to training, paying attention to skill acquisition and maintenance of skills
3. Delivery of intervention	Ensures interventions are delivered competently and to protocol throughout the study period
4. Receipt of intervention	Ensures intended interventions are actually received and understood by study participants
5. Enactment of intervention skills	Ensures study participants use intervention skills in real-life settings

Using this framework, the aim of this review is to assess treatment fidelity reporting in BCI RCTs in primary dental care settings to provide an overview of the use of fidelity strategies within the field, increase awareness of the importance of attention to fidelity within study design and conduct and make recommendations to dental researchers for fidelity strategy implementation in future research. We also aimed to compare the dental literature with other fields that have used similar review methods, namely general health behaviour trials [25] and behavioural tobacco cessation trials [26].

## Methods

### The eligibility criteria

We included RCTs of patient-level BCIs aimed at improving health outcomes that were delivered by dental team members in primary dental care settings. We defined these as settings where clinical dental treatment was provided by trained dental staff and so excluded any programmes involving BCIs such as oral health promotion delivered in community or other settings. We defined BCIs as ‘coordinated sets of activities designed to change specified behaviour patterns’ [27]. Where interventions were delivered by those who were not usual members of the dental team (i.e. by researchers or psychologists etc.), these studies were excluded. Included study designs were limited to RCTs. Articles in pre-print or published in journals without a peer-review process were excluded. Studies that were published in English language between 1 January 1980 and 1 August 2023 were included and inclusion was not limited by any particular outcome. The lower date threshold of 1980 was selected as it was anticipated that very few, if any, BCI RCTs would be published before this date. This review is reported in accordance with the Preferred Reporting Items for Systematic Reviews and Meta-Analyses extension for Scoping Reviews (PRISMA-ScR; Additional file 1) [28].

### Information sources and search strategy

The following electronic databases were searched: MEDLINE, PubMed, Scopus, PSYCInfo, CINAHL, AMED, Cochrane Central Register of Controlled Trials (CENTRAL), Dentistry and Oral Sciences Source and the NIHR Journals Library. An electronic search strategy was developed through discussion between authors and using keywords from relevant papers in the field. The search process was iterative and pilot searches were conducted to ensure an appropriate balance between specificity and sensitivity [29]. For example, after initial pilot searches, the term ‘behaviour change’ and its variants were removed from the strategy, as within the dental literature, this made the search too specific, risking the exclusion of eligible articles.

As this review aimed to include all information relating to intervention fidelity, we combined the electronic search with forward and backwards citation chasing [30]. This was conducted because reporting restraints such as low word-count limits have been cited as a potential barrier to fidelity reporting [31]. Such restrictions can result in reporting being split across multiple articles (e.g. results papers, papers about the development of interventions, papers outlining training procedures). Where more than one article was found relating to a BCI delivered in primary care dental settings, all articles were included. Some of these articles were not found through our electronic search strategy, and therefore, we carried out forward and backwards citation chasing for all included articles. Although, broadly speaking, the electronic search strategy was consistent between databases, minor tweaks were implemented to account for database style. The search strategy used in MEDLINE can be found at Additional file 2. The records extracted were managed in EndNote X9.2.

### Study selection

A screening tool was developed to standardise screening procedures, structured according to the Patient, Intervention, Comparator, Outcome, Study Type (PICOS) inclusion criteria (see Additional file 3). Titles and abstracts were screened by a team of four researchers, each screening a proportion of the articles. A random 20% sample was then double screened, and a disagreement rate of < 3% was found. Thereafter, full texts were retrieved and independently screened, and upon comparison a disagreement rate of < 5% was found. Whilst most conflicts were resolved through discussion, four studies were sent to a fifth reviewer (RH) for the final decision.

### Process of coding the articles for fidelity reporting

The NIH BCC checklist [23] was used to code the included articles. The checklist is designed to indicate the extent to which fidelity strategies were incorporated into a study by demonstrating the presence or absence of individual strategies that pertain to the five fidelity domains [23]. Each strategy that sits within a domain was evaluated separately. To assist with the standardisation of coding, a coding guide was developed and two coding reviewers (VL and RK) held regular coding calibration meetings. Each included study was coded independently as described by Borelli [23], coding each strategy in the checklist as present, absent but should be present or not applicable. When more than one article described the same intervention, these were read and coded together so that only one checklist was used per intervention. Once coding was completed independently, checklists were compared and discrepancies resolved through discussion. A high intercoder reliability rate of 88.6% was achieved, indicating confidence in coding reliability.

### Analysis of fidelity reporting using the checklist

To present data on individual fidelity strategies, the percentages of reported strategies were calculated as the ratio of the number of interventions for which the strategy was judged applicable (e.g. if an intervention was a one-off delivery, any fidelity strategies relevant to multi-session interventions would be coded as N/A). In this way, if a particular strategy was not judged applicable, it was removed from the calculation.

There is currently no consensus about how many of the NIH BCC fidelity strategies should be implemented for an intervention to be considered to have achieved a high level of fidelity [24]. Therefore, a conservative cut off was predefined whereby if 50% or more of the available strategies in any domain were reported, this was regarded as ‘good’ implementation of treatment fidelity strategies.

To provide an indication of the extent to which different fidelity domains were reported, mean proportions were calculated for each intervention by summing the number of present strategies and dividing by the number of strategies judged appropriate. Descriptive statistics were used to illustrate the extent to which fidelity domains were reported within the included interventions. In addition, descriptive statistics were used to draw comparisons between fidelity reporting in trials testing BCIs in primary care dental settings and two other fields, general health and tobacco cessation using reviews that have implemented similar methods [25, 26].

## Results

### Search results

**Fig. 1:**
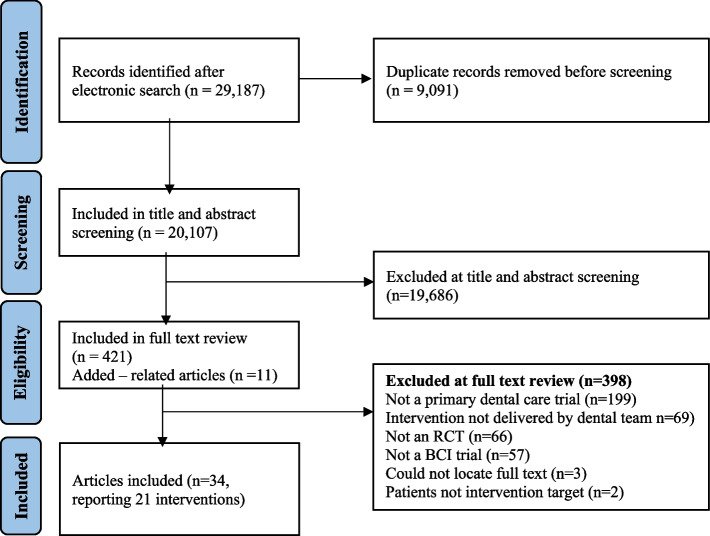
PRISMA flow diagram

The electronic search strategy identified 29,187 records. Once duplicates were removed 20,096 records remained. After title and abstract screening and excluding those not relevant to this review, 421 full texts were examined, and an additional 11 articles were identified through forward and backwards citation chasing. Of these, 398 were excluded, leaving 34 articles describing 21 BCIs to be included. Reasons for exclusion were recorded from the full text stage. These are depicted in Fig. 1.

### Summary of included studies

Characteristics of included articles are given in Table 1.

**Table 1:** Characteristics of included studies

No	First author	Year	Study title	Study design	Study participants	Intervention delivered by	No. interventionists	Control group overview	Intervention(s) overview	Primary outcome measure(s)	Results summary
1	Stevens V. J	1995	Making the most of a teachable moment: A smokeless-tobacco cessation intervention in the dental office	Two-arm RCT	576 male smokeless tobacco users; males aged 15 years +	Dental hygienists and dentists (for every intervention delivery)	Not reported	Usual care—patients may or may not receive advice to stop tobacco use, depending on personal practice habits of their dental care providers	Verbal education, goal setting, self-help written advice, video clips, personalised oral health feedback, direct unambiguous quit advice. Follow-up call to reiterate intervention, plus monthly tip sheet and newsletter	Self-report tobacco use; biochemical verification of tobacco use	At 3 months post intervention, a larger proportion of the IG self-reported abstinence from smokeless tobacco, also at 12 months
2	Hovell M. F	1996	An adolescent tobacco-use prevention trial in orthodontic offices	Two-arm cluster trial	16,915, 11 to 18 years old, with bands or braces	Orthodontist staff (unspecified)	Not reported	Usual care—no provision of anti-tobacco counselling, no alteration of offices in line with a ‘tobacco free’ direction	Minimal intervention comprising ‘tobacco-free office environment’. Delivery of anti-tobacco counselling, ‘prescriptions’ about tobacco related topics (teachable moment). Patients told not to start smoking	Self-report 30-day tobacco use; self-report use of any form of tobacco more than 100 times	30-day tobacco use was not sig. different in the CG vs IG 2 years post randomisation. There was no sig. difference between CG and IG in the use of tobacco
3	Severson H. H	1998	Using the hygiene visit to deliver a tobacco cessation program: results of a RCT	Three-arm cluster RCT + two-arm cluster RCT	4662 smokers, smokeless tobacco users, or both aged 15 years +	Dental hygienists	Not reported	Usual care—practitioners free to use clinical judgement as to whether to provide cessation advice	Persuasive communication, goal setting, goal review and behavioural education	Self-report abstinence from smoking products	No sig. results for cigarette smokers Smokeless tobacco users sig. more likely to quit in the IG vs CG. Sustained quit rate for CG was lower compared to the IG
	Andrews J. A	1999									
4	Curtis B	2007	Recruitment and standardization of a group of Australian dentists for a multi-practice study on dental caries prevention	Two-arm cluster RCT	902 dental patients (children and adults)	Dentists	22	Usual care	Mixed clinical and patient management intervention including some education-based behaviour change techniques (coaching around toothbrushing and goal setting re: diet)	DMFS increment, caries risk status	Caries risk was sig. reduced in the IG. Sig. incremental DMFS differences were shown between IG and CG at 2 and 3 years, favouring the IG, although end-point scores non-sig
	Curtis B	2008	The Monitor Practice programme: is a non-invasive management of dental caries in private practice effective?								
	Evans R	2008	The Caries Management System: an evidence-based preventive strategy for dental practitioners. Application for adults								
	Evans R	2016	The Caries Management System: are preventive effects sustained post clinical trial								
5	Gordon J. S	2007	The 5As vs 3As plus proactive Quitline referral in private practice dental offices: preliminary results	Three-arm cluster RCT	2177 tobacco-using dental patients	Dentists, dental hygienists, and dental assistants	Not reported	Usual care	One off brief counselling session 5As and 3As tailored tobacco interventions (with goal setting)	Self-report tobacco use in last 7 days; contact with a telephone Quitline	Tobacco quit rates *non-sig.* for both IGs. 3As more likely to have made a Quitline referral than 5As
6	Clarkson J	2009	How to influence patient oral hygiene behaviour effectively	Two-arm RCT (patient level) + two-arm cluster RCT	778 dentate adults	Newly qualified dentists	87	Usual care—possibly with oral hygiene advice as part of the appointment	Modelling, rehearsal of skills, planning implementation and education	Knowledge about oral health behaviours (brush timing, duration and method)	IGs both the individual-level RCT and the cluster RCT, behavioural outcomes were favourable
7	Gordon J	2010	Tobacco cessation via public dental clinics: Results of a RT	Two-arm cluster RCT	2549 adult (18 years +) smokers	Dentists, dental hygienists and dental assistants	Not reported	Usual care—practitioners delivered their usual tobacco cessation practices	5As tailored smoking cessation counselling (Ask, Advise, Assess, Assist, Arrange), plus pharmaceutical assistance (nicotine patches and lozenge), plus printed material, culturally tailored	Point prevalence (self-report tobacco use last 7 days); sustained abstinence (self-report time since last tobacco)	IG reported sig higher abstinence rates at follow-up for both point prevalence and prolonged abstinence
8	Hanoika T	2010	Intensive smoking-cessation intervention in the dental setting	Two-arm RCT	56 adult smokers	Dentists and dental hygienists	Not reported	No intervention provided	Behavioural and pharmaceutical approaches, pts counselled to set a quite date plus two regimes to prevent relapse. Self-help materials plus nicotine patches. Information concerning nicotine gum. Reinforcement of smoking cessation counselling	Smoking abstinence rate (level of cotinine present in saliva);	Abstinence rates at 3 months post-intervention were found to be higher in IG than CG (decreasing up to 1 year post-intervention)
9	Halvari A. E. M	2012	Self-determined motivational predictors of increases in dental behaviours, decreases in dental plaque, and improvement in oral health: a RCT	Two-arm RCT	158 university students	Dental hygienist	Not reported	Autonomy supportive standard care (authors state that due to ethical reasons, standard care included more autonomy support than in a true usual-care comparison)	Education, demonstration of effective brushing and flossing with practice and feedback, offering rationales for dental behaviours and how they mitigate disease, offering options concerning at home dental care routine (conducted in an autonomy supportive way)	Autonomy support and competence (self-report); autonomous motivation for oral home care (self-report); dental behaviours (self-report); clinical assessments	Those in IG positively affected perceived dental competence and dental behaviours and also promoted decreases in plaque and gingivitis. IG had higher perceived autonomy support compared to CG. Intervention did not yield a change in dental home care behaviours
	Halvari A. E. M	2019	Autonomy-supportive treatment, oral health-related eudaimonic well-being and oral health: a RCT	Two-arm RCT	138 university students					Autonomy support (self-report); eudaimonic well-being (self-report); clinical assessments	Intervention positively predicted perceived autonomy support measures, as well as increases in oral health-related behaviour goals and personal growth, and reductions in bacterial dental plaque and gingivitis
10	Neff J. A	2013	A brief motivational intervention for heavy alcohol use in dental practice settings: Rationale and development	Two-arm cluster RCT	103 adult alcohol drinkers who exceeded the National Institute on Alcohol Abuse and Alcoholism recommended drinking limits	Dental hygienists	Not reported	Usual care	Tailored risk information using MI spirit to reinforce preventive oral health behaviours and build self-efficacy. Use of verbal and pictorial information, and providing a comparison with a non-drinker	Alcohol consumption (self-report: total drinks consumed per week)	Sig. decreases in total drinks per week in both IG and CG at 3 months; at 6 months, the decreases in IG exceeded decreases in the CG No sig. findings in light or moderate drinkers
	Neff J. A	2014	Effectiveness of a screening and brief intervention protocol for heavy drinkers in dental practice: a cluster-randomised trial								
11	Virtanen S. E	2015	Evaluation of a brief counseling for tobacco cessation in dental clinics among Swedish smokers and snus users. A cluster RCT (the FRITT study)	Two-arm cluster RCT	467 adult dental patients currently using tobacco daily	Dentists or dental hygienists	54	Usual care provided by clinical for tobacco cessation (if any)	Structured brief advice based on the 5As. Provision of a leaflet	Total abstinence from tobacco during seven days preceding follow-up (self-report)	No sig. results between IG and CG on total tobacco abstinence (unadjusted analyses)
12	Lee M-Y	2017	Effect of an oral healthcare program on gingival health status in rural areas of South Korea	Two-arm RCT	42 middle-aged adults (40 years +)	Dental hygienists	Not reported	Active control: session of individual education plus provision of brochure and oral hygiene products	CG delivery plus education and professional oral hygiene sessions (8 weeks) about toothbrushing	Plaque index, gingivitis/periodontal disease (BoP score)	Plaque index was sig. decreased in the IG, and sig. differences between groups at each time point. Sig. decrease in BoP scores between CG and IG
13	Colvara B. C	2018	Motivational interviewing in preventing early childhood caries in primary healthcare: A community-based RCT	Two-arm cluster RCT	414 newborn children	Dental surgeons and dental hygienists	Not reported	Usual care—oral health protocol (recommend to undergo a dental visit in first year of life) without using motivational interviewing	Delivery of CG plus prevention information concerning early childhood caries using motivational interviewing techniques	DMFs	DMFS between groups was sig. different in favour of IG
	Faustino-Silva D. D	2019	Motivational interviewing effects on caries prevention in children differ by income: a randomised cluster trial								
	Faustino-Silva D. D	2019	Effectiveness of motivational interviewing training for primary care dentists and dental health technicians: results from a community clinical trial								
14	Ramsay C. R	2018	Improving the quality of dentistry (IQuaD): a cluster factorial RCT comparing the effectiveness and cost–benefit of oral hygiene advice and/or periodontal instrumentation with routine care for the prevention and management of periodontal disease in dentate adults attending dental primary care	Factorial cluster + individual RCT	1877 adult dental patients with either gingivitis or moderate periodontitis	Dentist and hygienists	Not reported	Usual care—routine oral hygiene advice as per current practice	Personalised oral hygiene advice focusing of self-efficacy (show, do, tell model), plus implementation intentions planning plus periodontal instrumentation (PI), PI at 6-month or 12-month intervals	BoP at the gingival margin, oral hygiene self-efficacy	No sig. differences in gingival bleeding between the no periodontal instrumentation, 6- or 12-month groups or between CG or IG
	Clarkson J	2021									
15	Hovell M	2018	A RCT of orthodontist-based brief advice to prevent child obesity	Two-arm cluster RCT	693 children aged between 8 and 16 years	Orthodontic staff (specific personnel not reported)	Not reported	Active control: three components as per intervention arm, but topic was about tobacco use/exposure	Health message ‘prescriptions’, personalised written goal and achievement tracking exercise around increasing physical activity and improving nutrition	BMI; consumption of junk food/fruit and veg items consumed in preceding 3 days (averaged); self-reported physical activity	No effects on PA. BMI decreased for males. Junk food consumption decreased over time. Fewer junk food items consumed in IG
16	Ntouva A	2015	Assessing the feasibility of screening and providing brief advice for alcohol misuse in general dental practice: A clustered RCT for the DART study	Two-arm cluster RCT	229 adult (18 +) dental patients who consumed above recommended levels of alcohol	Dentists	15	Active control: provision of a CRUK leaflet about mouth cancer prevention	Tailored brief advice about effects of alcohol, benefits of reducing alcohol, graph showing proportion of people in the UK who drink above and below recommended levels, practical advice for cutting down, standardised leaflets (NHS campaign). If a very high intake was detected, contact of local alcohol support services provided	Feasibility outcomes (not specified here) AUDIT tool score (alcohol misuse) at 6 months post	No sig differences were found between IG and CG in terms of AUDIT scores (although a positive trend shown)
	Ntouva A	2018	Evaluation of an alcohol screening and brief advice training programme for NHS general dental practitioners								
	Ntouva A	2019	Alcohol screening and brief advice in NHS general dental practices: A cluster randomized controlled feasibility trial								
17	Phetnin N	2020	Effectiveness of the oral health care program in older people with type 2 diabetes mellitus Muang District, Nakhan Ratchasima Province: A RCT	Two-arm cluster RCT	70 adults aged 60( +) with type 2 diabetes mellitus	Dentists and hygienists	Not reported	No intervention provided	Oral health education programme (including diabetes complications and relationship between diabetes and oral health), oral cleaning group practice, individualised oral hygiene instruction	Health Belief Model Scores; glycaemic status (HbA1c levels in blood), Oral Hygiene Index score	At follow-up sig. differences between IG and CG for all five domains (in favour of IG). Glycaemic status shown to improve in IG when compared with CG. Sig. differences at follow-up between IG and CG with IG demonstrating improved oral hygiene
18	Harris R	2020	Comparing how patients value and respond to information on risk given in three different forms during dental check-ups: the PREFER RCT	Three-arm RCT	Dentate adults (aged 18 +) at high or medium risk of poor oral health	Dentists	Not reported	Verbal information given on risk status (in usual way) + printed ‘key messages’ card about recommended health behaviour	Verbal information plus Traffic Light graphic card (RAG status); verbal information plus QLF image of patient’s teeth showing demineralisation or plaque (dentist discretion), with risk messages as per CG	Mean valuation of communication format (Willingness to Pay)	Patients willing to pay more for verbal information than other information formats. Sig. differences between verbal and traffic light and QLF and traffic light but no sig. differences between QLF and verbal information format
	Harris R	2020	Presenting patients with information on their oral health risk: the PREFER three-arm RCT and ethnography								
19	Holloway J. A	2020	RCT demonstrating the impact of behaviour change intervention provided by dental professionals to improve gingival health	Two-arm cluster RCT (plus power-toothbrush subset)	733 adult dental patients	Dental foundation dentists	Not reported	Usual care—based on undergraduate training—plus power toothbrush for subset	Goal setting, planning and self-monitoring of daily oral hygiene practices. Brushing instruction and demonstration with practice. Leaflet plus links to videos. High risk patients received gum health improvement agreement	BoP scores	BoP scores improved sig. in IG vs CG
20	Dimenas S. L	2021	A person-centred, theory-based, behavioural intervention programme for improved oral hygiene in adolescents: A randomized clinical field study	Two-arm RCT	312 adolescents aged 16–17 years	Dental hygienists	30	Usual care—oral health education information or instructions provided at one or several occasions	Based on cognitive behavioural theory and principles, goal setting, planning and self -monitoring (using diary) of oral hygiene habits, using a person-centred collaborative approach (inspired by MI)	MBI, oral hygiene behaviours	Sig. improvements in IG than CG. Sig. higher proportion of adolescents in IG brushed their teeth twice daily and cleaned interdentally ≥ 3 times a week than the CG. No sig. difference in time used for daily oral hygiene
21	Almabadi E. S	2021 (a)	The effect of a personalised oral health education program on periodontal health in an at-risk population: a RCT	Two-arm RCT	579 dental patients aged between 18 and 60	Oral health therapists/hygienists	2	Dental scaling and oral hygiene advice at a single appointment (no personalised oral health education program)	Personalised oral health education programme tailored to each patients’ oral health status and risk of periodontal disease. Use of MI, education, goals and plans, motivational communication around oral self-care + clinical restorative care if needed	PPD, BoP, confidence in oral care maintenance score	No sig. differences found on outcome between groups
	Almabadi E. S	2021 (b)	Reduction of hsCRP levels following an oral health education program combined with routine dental care		295 dental patients eligible for public dental services aged between 18 and 60					Systemic biomarkers of disease (hsCRP, lipid profiles and HbA1c)	Sig. difference between IG and CG in reduction of hsCRP, favouring the IG

*IG* intervention group, *CG* control group, *YO* years old, *Sig* statistically significant, *DMFS* decayed missing filled surfaces, *BoP* bleeding on probing, *HbA1C* glycated haemoglobin, *MBI* marginal bleeding index, *QLF* quantitative light-induced fluorescence, *PPD* periodontal probing depth, *hsCRP* high-sensitivity C-reactive protein

### RCT designs

The majority of included interventions were delivered using cluster RCT designs (*n* = 13), and one was delivered using both a cluster and factorial RCT design. The remainder of the interventions used parallel group RCT designs. One of the included studies was a feasibility study.

### Countries of origin

The majority of the included interventions were delivered in high income countries: United States of America (USA) (*n* = 7), United Kingdom (UK) (*n* = 5), Sweden (*n* = 2), Australia (*n* = 2), Norway (*n* = 1), South Korea (*n* = 1), Japan (*n* = 1), Thailand (*n* = 1) and Brazil (*n* = 1). Of the seven USA interventions, five concerned smoking cessation. In the UK, the second largest contributor, all of the included studies focused on oral hygiene prevention/improvement behaviours.

### Behavioural targets

Interventions targeted four behavioural areas with the most common being oral health behaviours (*n* = 11). These could be broken down into three groups: improvement/establishment of home dental care routines (e.g. tooth brushing, flossing, mouth rinses) (*n* = 9), improvement of diet related behaviours (e.g. sugar consumption) plus improvement/establishment of home dental care routines (*n* = 1) and dental visiting plus improvement/establishment of home dental care routines (*n* = 1). In addition, 7 interventions targeted tobacco use, 2 targeted alcohol use and 1 targeted obesity.

The behavioural interventions were all multi-component, often with several behaviour change approaches used in conjunction. Of the behaviour change approaches taken within the interventions, the most common were goal setting and planning (*n* = 13) and the provision of education/information about health consequences (*n* = 12). Five interventions incorporated rehearsal of practical skills (tooth brushing), 5 incorporated a counselling component, 4 used motivational interviewing (MI) techniques, 2 used modelling, 1 used a self-help style behavioural intervention, 1 incorporated a coaching style behavioural component, 1 used an autonomy-supportive approach, 1 altered environmental cues and 1 used persuasive communications.

### Interventionists

A range of dental team members, most commonly dentists (*n* = 14), and least commonly dental nurses (*n* = 2), delivered the interventions. Some studies did not report which specific team members delivered the interventions (*n* = 2). A large number of studies did not report how many individual dental team members delivered the interventions (*n* = 14), and for those that did report this (*n* = 7), a total of 210 dental team members were involved in delivering the BCIs.

### Control group characteristics

Most of the included studies used a ‘standard care’ approach (*n* = 15). Within these studies, control groups were most commonly described as treatment as usual where participants may or may not receive advice about the behavioural target, as would usually be provided in a standard dental appointment (e.g. smoking cessation advice, tooth brushing advice, diet advice). In terms of fidelity practices, control groups were often not well defined or measured. However, 6 of the included studies used an ‘active control’ in which the control group treatment was predefined and described. Only 1 group of studies [32–35] clearly defined the ‘standard care’ group by using a time and motion study to report exactly what standard care looked like in the study setting.

### Intervention fidelity strategy reporting

Nine of the RCTs mentioned fidelity explicitly in their articles (although none reported the use of any fidelity framework or guidance). Three of those stated that they either did not set out to measure fidelity due to implementation difficulties [36–40] or that despite attempting to measure and maintain strategies to improve fidelity, this proved difficult in the setting [41, 42]. The remaining 6 stated that they did attend to fidelity within their trials: [43–52]; however, only 1 of these [44] used 50% or more of the strategies recommended within each domain of the NIH BCC framework.

### Individual fidelity strategy reporting

The fidelity strategies reported in the included studies are summarised in Table 2.

**Table 2:** Percentage of studies reporting fidelity strategies, with comparison review data

**Checklist item description**	% of studies reporting intervention fidelity strategies					
	**Primary dental care BC trials**	**General health BC trials (1990–2000)**			**Smoking cessation BC trials**	
	%	*n*	%	*n*	%	*n*
**Study design domain**						
** Intervention condition information:**						
1a. Length of contact(s) (minutes)	57.1	21	90.0	329	59.3	755
1b. Number of contact(s)	76.2	21	98.0	331	76.0	755
1c. Content of intervention	85.7	21	95.0	341	88.0	755
1d. Duration of contacts over time	35.7	14	67.0	341	87.3	755
**Comparison condition information:**						
2a Length of contact(s) (minutes)	19.1	21	67.0	240	43.2	755
2b. Number of contact(s)	38.1	21	86.0	246	56.7	755
2c. Content of treatment	57.1	21	93.0	266	74.3	755
2d. Duration of contacts over time	27.2	11	90.0	252	73.1	755
2e. Method to ensure ‘dose’ is equivalent between conditions	16.7	6	NR	NR	NR	NR
2f. Method to ensure ‘dose’ is equivalent within conditions	33.3	21	NR	NR	NR	NR
3. Specification of interventionist credentials	19.1	21	69.0	293	63.1	690
**Theoretical model upon which the intervention is based articulated:**						
4a. Active ingredients specified and incorporated in the intervention	71.4	21	74.0	338	62.4	755
4b. Use of experts or protocol review group to determine whether the intervention protocol reflects to underlying theory/guidelines	9.5	21	NR	NR	NR	NR
4c. Plan to ensure measures reflect theoretical constructs/mechanisms of action	57.1	21	NR	NR	NR	NR
5. Potential confounders identified and discussed	52.4	21	NR	NR	NR	NR
6. Plan to address possible setbacks in implementation articulated	14.3	21	NR	NR	NR	NR
7. If more than one intervention, all described equally well	60.0	10	NR	NR	NR	NR
**Training interventionists domain**						
1.Description of how interventionists were trained	57.1	21	29.0	291	23.3	689
2. Standardisation of interventionist training	38.1	21	29.0	291	21.3	689
3. Assessment of interventionist skill acquisition	23.8	21	18.0	292	7.4	689
4. Assessment and monitoring of interventionists skill maintenance	23.8	21	27.0	288	24.7	689
5. Interventionist characteristics sought/avoided articulated a priori	9.5	21	NR	NR	NR	NR
6. At the interventionist hiring stage, assessment of whether or not there is a good fit between the interventionist and intervention	14.3	21	NR	NR	NR	NR
7. Training plan considers different learning styles and education levels	42.9	21	NR	NR	NR	NR
**Delivery of intervention domain**						
1.Mehod to ensure content of the intervention delivered as intended	19.1	21	51.0	332	24.2	755
2. Method to ensure dose of the intervention delivered as intended	23.8	21	34.0	322	15.2	755
3. Mechanism to assess if interventionist actually adhered to intervention plan	33.3	21	30.0	287	21.1	689
4. Assessment of nonspecific treatment effects	0.0	21	7.0	283	2.6	688
5. Use of a treatment (intervention) manual	38.1	21	38.0	301	32.1	755
6. A plan for the assessment of whether or not the active ingredients were delivered	9.5	21	NR	NR	16.4	755
7. A plan for the assessment of whether or not proscribed components were delivered	0.0	21	NR	NR	7.8	700
8. A plan for how contamination between conditions will be prevented	66.7	21	NR	NR	12.4	755
9. A priori specification of treatment fidelity	4.8	21	NR	NR	10.9	755
**Receipt of intervention domain**						
1.Assessment of the degree to which participants understood the intervention	19.1	21	45.0	332	6.6	755
2. Strategies to improve participant comprehension of intervention	14.3	21	57.0	331	5.2	755
3. Participants’ ability to perform intervention skills assessed during intervention period	25.0	20	54.0	326	44.2	755
4. Strategy to improve subject performance of intervention skills during intervention period	36.8	20	58.0	325	9.8	755
5. Multicultural factors considered in development and delivery of intervention	19.1	21	NR	NR	13.8	755
**Enactment of intervention skills domain**						
1. Participant performance of intervention skills assessed in settings in which the intervention might be applied	33.3	20	73.0	330	43.0	755
2. A strategy to assess performance of intervention skills in settings in which the intervention might be applied	14.3	20	50.0	327	6.6	755

Large ranges in fidelity strategy reporting were found. The largest range was observed in the domain of *study design*, where only 9.5% (2/21) of the studies reported using experts or a protocol review group to determine whether intervention protocols reflected their underlying theories, whereas 85.7% (18/21) reported the content of the intervention. In the *training* domain, reporting ranged from only 9.5% (2/21) of the studies articulating a priori what characteristics were sought in their interventionists to 57.1% (12/21) providing a description of how interventionists were trained. A large range was also seen in the *delivery* domain with 0 studies reporting on either the assessment of nonspecific treatment affects, or the provision of a plan for the assessment of proscribed (unhelpful) components, whereas 66.7% (14/21) reported a plan for preventing contamination between study arms (albeit a large number of these were attended to through cluster trial designs). Similarly, within the domain of *receipt*, 14.3% (3/21) of the studies were found to have specified a strategy to improve participant comprehension of interventions, and 36.8% (7/19) had implemented a strategy to improve participants’ performance of intervention skills during the intervention period.

The three most commonly reported fidelity strategies were: detailing the content of the intervention (85.7%, 18/21), reporting the number of intervention contacts (76.2%, 16/21), and specifying the theoretical ‘active ingredients’ of interventions (71.4%, 15/21), all of which are within *study design*. The three least commonly reported fidelity strategies were an assessment of non-specific treatment effects (0%), a plan for whether or not proscribed components were delivered (0%) and a priori specification of treatment fidelity (4.8%, 1/21), all of which are within *delivery*.

### Domain specific fidelity strategies

*Study design* was the domain most frequently reported, with seven studies reporting at least 50% of the appropriate checklist items and all studies reporting at least three of the *design* strategies. The least reported domain was *delivery*, with only two studies reporting at least 50% of the domain strategies and three reporting no *delivery* strategies at all. Additionally, three studies did not report any *receipt* strategies and 13 of the studies did not report any *enactment* strategies. The full domain breakdown can be found in Table 3.

**Table 3:** Percentages of studies reporting 50% or more fidelity strategies and those not reporting any strategies

	Design domain	Training domain	Delivery domain	Receipt domain	Enactment domain
% of studies reporting at least 50% of strategies within domain	33% (7/21)	24% (5/21)	5% (1/21)	14% (3/21)	35% (7/20)
% of studies not reporting any strategies within domain	0%	29% (6/21)	14% (3/21)	38% (8/21)	65% (13/20)

All included studies reported fidelity strategies that were in at least two of the five NIH BCC domains. However, only four (19%) reported at least one strategy in all five domains, seven (33%) reported at least one strategy in four domains, six (29%) reported at least one strategy in three domains and four (19%) reported at least one strategy in two domains. Only one study [44], an MI-inspired oral health education programme delivered to adolescent dental patients, reported over 50% of the appropriate fidelity strategies in all domains.

There were, however, stand-out examples where high-fidelity reporting (defined as reporting 80% or more of the relevant strategies [25]) within a domain was found. For example, for *study design*, Neff et al. [50, 51] reported 80% of the strategies; for *training*, Colvara et al. and Faustino-Silva et al. [36–38] reported 86% of the strategies, as did Ntouva et al. [47–49]; for *receipt*, Clarkson et al. [53] reported 80% of the strategies and Dimenäs et al. [44] reported 100% of the strategies; and finally for *enactment*, three studies reported 100% of the relevant strategies [44, 54, 55]. No studies met the ‘high-fidelity’ threshold for *delivery*.

To provide an overview of the amount of fidelity reporting present within the literature, and to show which areas of fidelity are reported, Table 4 shows the mean proportion of reported fidelity strategies within each domain.

**Table 4:** Mean proportion of reported fidelity strategies within each domain

NIH BCC fidelity domain	Mean proportion (SD)	*n*
Study design	0.45 (0.17)	21
Training	0.29 (0.27)	21
Delivery	0.21 (0.15)	21
Receipt	0.24 (0.41)	21
Enactment	0.28 (0.28)	20

### Comparisons between primary dental care research and other fields

Table 2 compares the prevalence of fidelity reporting within dental trials to that in other fields (general health behaviour trials and smoking cessation trials) using similar methods.

When comparing the proportions of reported fidelity strategies, it is clear that general health behaviour change trials reported the most checklist strategies. In nine out of ten of the strategies within *study design*, the highest reporting proportions could be seen in the health behaviour change literature. Similarly, in both *receipt* and *enactment*, all of the strategies had higher reporting proportions in the health behaviour change literature.

However, primary dental care trials had higher reporting proportions for three of the four strategies within the *training* domain.

Similarities across reviews were seen in the most commonly reported strategies. For example, high proportions could be seen within all three reviews for *study design* strategies of reporting intervention content (85.7% primary dental care trials, 95.0% general health behaviour change trials, 88.0% smoking cessation behaviour change trials), reporting of active ingredients of interventions (71.4% primary dental care trials, 74.0% general health behaviour change trials, 62.4% smoking cessation trials) and recording the number of intervention contacts (76.2% primary dental care trials, 98.0% general health behaviour change trials, 76.0% smoking cessation trials).

There were also similarities between strategies that had low reporting proportionality. For example, for *delivery*, low reporting was found for a strategy to assess non-specific treatment effects (0% for primary dental care trials, 7% for general health behaviour trials and 2.6% for smoking cessation trials).

Overall, more fidelity reporting was evidenced in *study design* across all three reviews than the other domains, and generally lower reporting could be seen for *receipt* strategies.

### Fidelity monitoring methods

In terms of the reported methods used to monitor fidelity in primary dental care trials, interventionist self-reports were used in 2 studies [43, 47–49], and participant self-reports were used in 3 studies [50, 51, 56–58]. One study used a combination of interventionist and participant self-report methods [55].

Objective measures such as the use of voice recordings or numbers of Quitline referrals (for smoking trials) were used in 3 of the studies [45, 46, 52, 59]. One study used a combination of interventionist self-report and an objective measure [44], and one study used a combination of participant self-report and an objective measure [54]. Ten (48%) studies did not report fidelity monitoring methods.

## Discussion

This review examined the reporting adequacy of treatment fidelity in RCTs testing BCIs in primary dental care settings according to the NIH BCC fidelity framework. To the best of the authors’ knowledge, this is the first review to explore treatment fidelity practices within this field. Enhancing fidelity practices is an essential step in the development of a reliable evidence base, with the ultimate goal of translating research findings into real-world practice.

As the NIH BCC fidelity framework positions fidelity in discrete domains, we set out our discussion using this format.

### Study design

Our results indicate that researchers testing BCIs in primary dental care typically implement and report fidelity strategies that relate to the intervention condition(s), such as information about intervention dose and content, but that far less attention is given to control conditions. Our findings suggest control group descriptions are often lacking in detail or simply state ‘usual care’. The comparisons laid out in this review between primary dental care research, general behaviour change trials [25] and tobacco cessation research [26] suggest that this problem is more common in primary dental care research. It is important to properly consider and report the implications of control conditions in trials, as failure to do so allows uncertainties to arise about intervention effects. Our finding feeds into a wider debate around usual care comparators used within pragmatic trials of complex health interventions where it has been found that consideration of the context or content of a usual care arm is lacking [60]. However, our review identified one exemplar study [32] in which multiple fidelity strategies were reported when defining the ‘usual care’ group, and this approach could serve as a guide for how to enhance fidelity practices in usual care arms.

### Training of interventionists

Our review also revealed that the training domain was generally reported more commonly in the primary dental care literature than in the comparison reviews. When examining this domain at the strategy level, we found that approximately 57% of the dental studies reported a description of how interventionists were trained (compared to 29% and 23% in the comparison reviews), suggesting an awareness of the importance of good training practices within dental trials. However, our review also revealed that only approximately 24% of the dental trials reported on both implementing strategies to assess interventionist skill acquisition and monitoring of skill drift over time, and only approximately 10% reported the characteristics of interventionists that are sought or to be avoided. This strategy-level reporting disparity demonstrates that whilst training is being considered by primary dental care researchers, there has been limited adoption of strategies to enhance the longer-term effects of training and interventionist selection. A recently published feasibility study conducted in primary dental care provided some discussion about the appropriateness of interventionists and how training can be used to bridge gaps and bring all interventionists up to the same skill levels, whilst acknowledging that the resources required might be considerable [61].

When conducting trials in busy real-life dental settings, often alongside ‘business as usual’ and in environments with competing demands upon staff, it is important to prevent skills drift and check that training achieves its objectives. When primary dental care sites are involved in research, resource issues such as limitations in appropriate space for research, staff availability/staff with requisite experience or time pressures [62] can mean that fidelity strategies such as ‘ensuring a good fit between the interventionist and intervention’ or involving interventionists with predefined ‘sought-after’ characteristics are simply not viable options available to researchers. Possible solutions could include working with interventionists to increase buy-in to an intervention, or it could even mean making the decision to stop working with an interventionist if it is clear that there is a problem with an individual’s ‘fit’ with an intervention. Training fidelity strategies are wholly within the control of the research team to implement, and this review has demonstrated a need for more consideration to be given to strategies to improve training practices within primary dental care BCI trials. Our review identified a few stand-out exemplars that achieved high fidelity scores in training [36, 49], and these could serve as a guide for primary dental care trialists.

### Intervention delivery

Our findings demonstrated that delivery was the most poorly reported fidelity domain. This is evidenced by the domain mean proportion score of 0.21, together with the finding that only 5% (1/21) of the included studies incorporated 50% or more of the strategies that pertain to delivery. Poor delivery reporting was also found in the two comparison reviews. This finding appears to contradict previous research indicating that delivery fidelity is usually the best understood [63] and reported within various fields of research [24, 64, 65]. In addition, two recent reviews that examined delivery fidelity in behaviour change trials delivered by healthcare workers in real-life settings reported fidelity rates of 57% [66] and 48% [67]. Therefore, it appears that there are disparities across fields.

It is possible that poor reporting could be linked to time constraints of dental professionals [62, 68]. Looking at examples of delivery strategies implemented by the only trial within this review that adopted over 50% of the strategies within the domain of delivery, it is clear that these strategies could be time consuming and burdensome for interventionists. When setting up and managing trials, researchers need to make decisions that balance burden and study conduct. When slotting research practices into ‘business as usual’ healthcare environments, particular attention should be given to minimising process burden (interventionist and participant burden) wherever possible. One solution could be that the ‘fidelity burden’ is transferred from interventionists to research teams, with the onus of fidelity data collection, and associated assessment always with the researchers.

### Intervention receipt

This review has also demonstrated poor reporting of fidelity strategies within the receipt domain. Reporting levels appear mixed in the comparison reviews, with general behaviour change trials reporting slightly higher proportions of strategies than in either dental or smoking cessation trials. Within the primary dental care literature, the most commonly reported checklist item (40% of the included studies) was a strategy to improve participant performance of intervention skills during the intervention period. Interestingly, the two studies that reported 50% or more of the recommended strategies for receipt had those strategies embedded within the intervention itself, rather than as additional processes. Other studies paid no attention to this domain, with 38% of the included studies not implementing any receipt strategies. This finding is in line with previous research showing that receipt is poorly defined and reported in health intervention research [69].

There are two dominant schools of thought that feed into the approach taken by researchers when conceptualising fidelity assessment which may help to explain low reporting in this domain: (1) that the domain of receipt (and incidentally, enactment) is seen as a *moderator* of fidelity, thus treating participants more as passive recipients of interventions [70], and (2) as per the NIH BCC framework, where receipt is a *component* of fidelity and participants are seen as taking an active role in how an intervention works. It is possible that the domain of receipt within the dental trials has been conceptualised in a way that frames participants as passive recipients of interventions, resulting in a paucity of strategies pertaining to assessing and enhancing receipt fidelity.

### Intervention enactment

Finally, our review shows that *enactment* reporting is inconsistent, with a large proportion of the studies not reporting any enactment strategies (65%) and approximately one third reporting 100% of the suggested strategies. The comparison reviews found more studies reported enactment enhancing strategies (73% and 43% respectively), but fewer reported enactment monitoring strategies (50% and ~ 7%, respectively), a pattern echoed in dental trials. Other research has shown that, generally, enactment is not well attended to in a variety of fields, leading to a general consensus that improvements are needed to enhance enactment fidelity practices [65, 71].

In addition to the argument mentioned within the delivery section, that enactment may be seen as a moderator rather than a component of fidelity [70], one further reason for reporting problems in this domain could be difficulties with operationalising what enactment would actually mean within any given study. Enactment is conceptually complex [72, 73]. An interesting point posed by Ginsburg and colleagues in their paper which sought to provide guidelines on how to practically and comprehensively assess fidelity in complex group-level interventions suggested that it could be difficult to categorise (and therefore meaningfully implement and measure) even seemingly straightforward intervention components into discrete fidelity domains [74]. This concurs with our findings and an example is presented in Dimenäs and colleagues’ article [44] where participant diaries were used as a component of the intervention as a means to record at-home oral hygiene behaviours (the behavioural target). The use of diaries in this way could tick delivery, receipt and enactment boxes according to the NIH BCC checklist. Ginsburg and colleagues further point out that there is little empirical work that explores how researchers should approach operationalising delivery, receipt and enactment as distinct elements or even if they could be combined and meaningfully linked to improvements in intervention effectiveness [74]. This conceptualisation complexity could explain why strategies pertaining to the domain of enactment are omitted from many of the trials examined as part of this review. Our stance, however, is that enactment is an important component of fidelity, as participants in research are active players and that efforts should be made to think through the operationalisation of enactment strategies.

### Recommendations

There are multiple studies that provide recommendations for how to enhance and assess fidelity within the literature across various fields [24, 26, 74]. Due to the low fidelity reporting in primary dental care BCI trials and the lack of evidence for the use of any frameworks or guidelines to help embed fidelity strategies, we suggest the following focus for improvement:

*Recommendation 1*: Primary dental care trial investigators should be familiar with fidelity concepts, and the NIH BCC framework provides an accessible starting-point on how to conceptualise, implement and report fidelity in behaviour change research.

*Recommendation 2*: Fidelity should be conceptualised and operationalised by investigators from study inception (at the funding application stage ideally) with a willingness to be flexible in approach to accommodate the challenging setting of primary dental care.

*Recommendation 3*: As the primary dental care setting is a challenging context within which to conduct trials, ‘fidelity burden’ should be the responsibility of research teams, and appropriate methods built in to achieve this.

*Recommendation 4*: A focus on objective fidelity monitoring methods should be adopted, recognising ethical considerations (i.e. use of audio or video recordings).

Primary dental care trialists may be unfamiliar with the concept of fidelity or what appropriate strategies to implement and report. Following the useful example by Salloum and colleagues in their review of behavioural tobacco treatment trials [26], Table 5 provides context specific examples of how to implement and report fidelity strategies to assist researchers in fidelity planning and implementation.

**Table 5:** NIH BCC checklist with relevant primary dental care examples

NIH BCC treatment fidelity strategy	Examples relevant to primary dental care trials
**Study design**	
1. Treatment cost in the intervention condition(s) a. Length of session(s)	10-min oral health coaching session
b. Number of contacts	3 sessions, 2 weeks apart
c. Content of treatment	MI approach (specify which elements of MI used) focused on at home toothbrushing behaviours, including practice, feedback and implementation intention goal setting and reinforcement/revisiting over sessions
d. Duration of contact over time	30-min over three sessions, 2 weeks apart
2. Treatment dose in the control or comparison condition a. Length of contact session	10-min
b. Number of contacts	1 session
c. Content of treatment	Standardised usual care: general toothbrushing and diet advice as per current guidelines following a standardised approach
d. Duration of contact over time	10-min single session
e. Method to ensure dose is equivalent between conditions	Only applicable if two interventions are tested. Methods include audio-recording interventions, in vitro observations to record timings
f. Method to ensure dose is equivalent within conditions	Video/audio recording interventions, computer metadata for timings (online interventions), subjective measures such as interventionists recording after each session (less reliable)
3. Specification of interventionist credentials	e.g. certificate in oral health education, 5 years plus experience in patient facing role
4. Report theoretical model or clinical guidelines	Motivational Interviewing, Social Identity Theory, ‘Delivering better oral health’ etc
5. Use of experts or protocol review panel to determine whether the intervention reflects underlying theory/guidelines	Optimally, experts outside of the research group to blind code intervention content to determine theory/guideline upon which intervention is based
6. Present potential confounders that limit the ability to make conclusions	Introduction of new dental team training that may alter dental team’s interactions with patients around oral health advice outside study procedures
7. Plans to address possible setbacks to implementation	Training plans to address staff turnover
8. If more than one intervention, all described equally well	Ensure descriptions are reported
**Training interventionists**	
1. Description of how interventionists were trained	Individual training session, group training sessions, training location, number of training hours, different types of training provided
2. Standardisation of interventionist training	Training materials consistent, training undertaken by same trainers, trainers all received consistent training (train the trainers’ model)
3. Monitoring of interventionist skill acquisition	Observations, role plays, written exam, discussion and feedback, coaching
4. Monitoring of interventionist skill maintenance	Audio recordings of intervention delivery sessions, in vitro observations, supervision, reflective practice, interviews with interventionists
5. Interventionist characteristics sought/avoided articulated a priori	e.g. confidence in talking to patients, oral good communication skills, warmth, computer skills
6. At the interventionist ‘hiring’ stage, assessment of whether or not there is a good fit between the interventionist and intervention	e.g. for a talking intervention, ensuring interventionist is confident with oral communication. For an intervention with children, someone with experience working with children. Additionally, ensure there is belief and buy-in to the intervention. Be prepared to stop if fit is problematic
7. Training plan considers different learning styles and education levels	Variety of training types (didactic, role play, discussions, visual aids, ‘on the job’ shadowing). Allowing for flexibility in training plans to suit the needs of individuals. Being prepared to provide more training to some than others, whilst being consistent with training content
**Delivery of intervention**	
1. Method to ensure content of the intervention delivered as intended	Optimally review of audio/video recordings of intervention sessions
2. Method to ensure dose of intervention delivered as intended	Optimally review of audio/video recordings to ensure accurate dose measurement. Could also use timing meta data for online interventions
3. Mechanism to assess if the interventionist actually adhered to the intervention	Review of audio/video recordings
4. Assessment of nonspecific treatment effects	Review of audio/video recordings, participant exit interviews, participant exit questionnaire completion, interventionist self-report (multi-methods would be optimal here)
5. Use of treatment manual	This could also be a check-list, crib-sheet, etc. Report that this was used in published reports
6. Assessment of whether or not the active ingredients were delivered	Optimally, review of audio/video recordings. Could also be check-lists or participant exit interviews
7. Assessment of whether or not proscribed components were delivered	Review of audio/video recordings of intervention sessions
8. A plan for how contamination between conditions will be prevented	Different emphasis and methods for different trial designs. Cluster trials, less emphasis needed. Parallel group designs need to monitor (record or observe) both control and intervention arms
9. A priori specification of treatment fidelity	All active ingredients to be delivered to every participant, with minimum competency ratings needed of 80%, judged by trained fidelity coders of recorded delivery sessions
**Receipt of intervention**	
1. Assessment of the degree to which participants understood the intervention	Standardised questions designed to check participant understanding built into intervention delivery
2. Strategy to improve participant comprehension of intervention	Use of videos to deliver information, with interventionist checking understanding throughout
3. Participants’ ability to perform intervention skills assessed during intervention period	Demonstration of toothbrushing technique by participant to interventionist with feedback
4. Strategy used to improve subject performance of intervention skills during intervention period	Confidence in ability to perform skill rated as part of intervention delivery, with coaching element built in designed to improve skills performance
5. Multicultural factors considered in development and delivery of intervention	Using appropriate and thorough patient and public involvement to meaningfully tailor interventions to target audiences
**Enactment**	
1. Participant performance of intervention skills assessed in settings in which the intervention might be applied	Use of diaries to review skills behaviour for multi-session interventions. Follow-up telephone calls to check intervention skill enactment for single session interventions
2. A strategy used to assess performance of the intervention skills in setting which the intervention might be applied	Text message reminders to perform intervention skills

There are several limitations to this review. First, as previously reported [75], defining primary dental care settings was challenging, and it is plausible that some studies were missed due to variations in setting reporting within the literature. Second, we applied a fidelity checklist to trials where the focus was not fidelity reporting. This may have resulted in lower fidelity scores being represented in this review. However, every effort was made to include all information published on the studies, and from our search, no explicit fidelity explorations were identified in primary dental care BCI trials. Third, due to a paucity of information reported in some of the articles, the fidelity coders found applying the checklist challenging at times. Despite this, a satisfactory interrater reliability score was achieved with the help of a period of ‘coding calibration’ at the start of the coding process supplemented by frequent open dialogue about coding processes among the team. Finally, we acknowledge that by limiting inclusion to English-only articles, this may have introduced bias, or this may have excluded some articles that would have otherwise met the inclusion criteria for this review. This was however, a necessary decision due to resource constraints.

## Conclusions

In conclusion, this scoping review revealed limited evidence of fidelity reporting within the primary dental care BCI trials literature. Inconsistent reporting was also found between studies demonstrating a lack of standardisation in approach. We have proposed recommendations to raise awareness of the methodological importance of considering fidelity strategies early on in the study design process, and we have identified exemplars within the literature that could be used as guides for future research in this complex setting. The implementation of well-thought-out fidelity plans has the potential to improve the reliability and reproducibility of trials testing BCIs in primary dental care settings, fostering improvements in the evidence base from which real-world interventions are drawn.

## Supplementary Information

Additional file 1: PRISMA-ScR. Completed PRISMA-ScR checklist.

Additional file 2: MEDLINE search strategy. The full MEDLINE electronic search strategy.

Additional file 3: PICOS Screening Tool. The screening tool used by the researchers involved in the title and abstract screening stage.

## Data Availability

All data generated or analysed during this study are included in this published article and its additional files.

## Abbreviations

NIH BCC: National Institutes of Health Behaviour Change Consortium
BCI: Behaviour change intervention
RCT: Randomised controlled trial
MI: Motivational interviewing
