# Supplementary material for: Scoping review of fidelity strategies used in behaviour change trials delivered in primary dental care settings
Source: Trials. 2024 Dec 18;25:824. doi: 10.1186/s13063-024-08659-9 (PMC11653899; doi:10.1186/s13063-024-08659-9)
Supplement: Supplementary file 2 — Additional file 2: MEDLINE search strategy. The full MEDLINE electronic search strategy. [file 13063_2024_8659_MOESM2_ESM.docx]

**MEDLINE Search Strategy**

| (Primary healthcare[MeSH] OR general dental practice[tiab], dental[tiab] or group practice[tiab], OR primary health care[MeSH] OR primary care[MeSH} OR primary care setting[tiab] or dental practice*[tiab]) |
| --- |
| **AND** |
| (random* controlled trial[MeSH] OR clinical trial[tiab] OR controlled trial[tiab] OR pragmatic trial[tiab] or trial[tiab] OR feasibility studies[tiab] or feasibility[tiab] OR pilot project[tiab] OR pilot study[tiab] or intervention[tiab]) |
| **AND** |
| Oral Health[MeSH] OR Dental Clinics[MeSH] oral hygiene[tiab] OR toothbrushing[tiab] OR dental health education[tiab] OR public health dentistry[tiab] OR community dental care[tiab] OR special care dentistry[tiab] OR paediatric dentistry[tiab] OR orthodontics[tiab] OR dentistry[MeSH] OR preventative dentistry[tiab] OR dental research[tiab] OR evidence-based dentistry[tiab OR geriatric dentistry[tiab] |
