# Supplementary material for: Scoping review of fidelity strategies used in behaviour change trials delivered in primary dental care settings
Source: Trials. 2024 Dec 18;25:824. doi: 10.1186/s13063-024-08659-9 (PMC11653899; doi:10.1186/s13063-024-08659-9)
Supplement: Supplementary file 3 — Additional file 3: PICOS Screening Tool. The screening tool used by the researchers involved in the title and abstract screening stage. [file 13063_2024_8659_MOESM3_ESM.docx]

**PICOS Screening Tool**

For an article to be included in the review, it must meet all of the inclusion criteria set out below.

| **PICOs** | **Include Description** | **Meets include description** | **Does not meet include description** |
| --- | --- | --- | --- |
| **Population** | Any dental patient, of any age |  |  |
| **Intervention** | Any behaviour change intervention (a coordinated set of activities designed to change specified behaviour) delivered by dental teams to patients. This could include clinical trials with pharmacological/ medical device elements, as long as one component of the intervention could be classed as a behaviour change intervention (i.e. oral health education) |  |  |
| **Control** | Any control. This could be standard care, or another intervention arm comparison |  |  |
| **Outcomes** | Any outcomes |  |  |
| **Study type** | Randomised controlled trials (any design), feasibility randomised controlled trials, pilot randomised controlled trials conducted in primary dental care settings (i.e. in settings where clinical dental treatment is usually provided by trained dental staff) |  |  |
